# Design of the Weight-loss Endoscopy Trial (WET): a multi-center, randomized, controlled trial comparing weight loss in endoscopically implanted duodenal-jejunal bypass liners vs. intragastric balloons vs. a sham procedure

**DOI:** 10.1186/s12876-018-0838-3

**Published:** 2018-07-18

**Authors:** Marcus Hollenbach, Christiane Prettin, Felix Gundling, Wolfgang Schepp, Jochen Seufert, Jürgen Stein, Thomas Rösch, Jens Aberle, Jürgen Feisthammel, David Petroff, Albrecht Hoffmeister

**Affiliations:** 1 Department of Medicine, Neurology and Dermatology; Division of Gastroenterology and Rheumatology, University of Leipzig, Liebigstrasse 20, D-04103 Leipzig, Germany; 2 Clinical Trial Center Leipzig, University of Leipzig, Leipzig, Germany; 3 Clinic for Gastroenterology, Hepatology and Gastrointestinal Oncology; Bogenhausen Clinic, Munich, Germany; 4 Clinic for Internal Medicine II; Division of Endocrinology and Diabetology, University of Freiburg, Freiburg, Germany; 5 Clinic for Internal Medicine; Division of Gastroenterology; Sachsenhausen Clinic, Frankfurt, Germany; 6 Clinic for Interdisciplinary Endoscopy; Center for Radiology and Endoscopy, University of Hamburg-Eppendorf, Hamburg, Germany; 7 Clinic for Endocrinology, Diabetology, Adiopsity and Lipids, University of Hamburg- Eppendorf, Hamburg, Germany

**Keywords:** Obesity, Endoscopy, Diabetes, Weight loss, Intragastric balloon, Bypass liner

## Abstract

**Background:**

Obesity is a global problem leading to reduced life expectancy, cardiovascular diseases, diabetes and many types of cancer. Even people willing to accept treatment only achieve a mean weight loss of about 5 kg using commercial weight loss programs. Surgical interventions, e.g. sleeve gastrectomy or gastric bypass are effective but accompanied by risk of serious complications and side effects. Less invasive endoscopic procedures mainly comprise the intragastric balloon (IB) and the duodenal-jejunal bypass liner (DJBL). To date, a randomized comparison between these devices has not been undertaken or shown to be superior to a sham procedure.

**Methods:**

We designed a multi-center, randomized, patient and assessor-blinded, controlled trial comparing weight loss in endoscopically implanted IB vs. DJBL vs. a sham procedure. A total of 150 patients with a BMI > 35 kg/m^2^ or > 30 with obesity-related comorbidities and indication for proton pump inhibitors are randomized to receive either IB, DJBL or a sham gastroscopy (2:2:1 ratio). All participants undergo regular dietary consultation. The IB will be removed after 6 months, whereas the DJBL will be explanted after 12 months. All patients will receive gastroscopies at implantation and explantation of the devices or sedation without gastroscopy to maintain blinding. Main exclusion criteria are malignant diseases, peptic ulcer or previous bariatric intervention. Weight loss 12 months after explantation of the devices, changes in comorbidities, quality of life, complication rates and safety will be evaluated.

**Discussion:**

This trial could help to identify the most effective and safest endoscopic device, thus determining the new standard procedure for endoscopic bariatric treatment.

**Trial registration:**

16th January 2017. DRKS00011036. Funded by the German Research Foundation (DFG).

## Background

Obesity is a growing global problem and an estimated 312 million people worldwide are affected [1]. Many studies have shown reduced life expectancy, higher risk of cardiovascular, pulmonary and gastrointestinal diseases, diabetes and many types of cancer to be associated with obesity [2]. Managing obesity is often disappointing as lifestyle alterations are an important, but frequently inadequate intervention. Although even moderate weight loss of 5–10% of body weight is associated with a meaningful improvement in insulin resistance, blood pressure and dyslipidemia [2, 3], only 20% of obese people are willing to accept treatment [1]. In addition, conservative treatment results only in mean weight loss of about 5 kg after one year using commercial weight loss programs and less than 2.5 kg using standard care. Moreover, only two thirds complete their respective programs even in the study setting [4].

Interventional surgical options comprise mostly sleeve gastrectomy and gastric bypass. These are effective but irreversible and associated with risk of serious perioperative complications, malabsorption and necessity of dietary supplementation. Furthermore, gastrointestinal symptoms such as reflux or vomiting occur in up to 20% of cases and in more than 10% of patients repeated surgery is required [5, 6]. Thus, many patients are unwilling to undergo this far-reaching step, and limited resources restrict surgical interventions to a small proportion of potential candidates.

In contrast, bariatric endoscopic procedures are reversible, minimally invasive, less costly and may offer a potentially lower risk approach compared to bariatric surgery [7]. These endoscopic approaches limit oral food intake, gastric exclusion or evoke malabsorption by partially inhibiting the breakdown or absorption of nutrients [8]. Intragastric balloons (IB) have been used for over 30 years and have been evaluated in many studies. They promote weight loss by physically decreasing intragastric volume and increasing gastric empting time. IB is usually removed after 6 months although recent devices allow implantation for up to 12 months [9]. Repeated implantations or implementation as an initial therapy prior to bariatric surgery are also feasible [10]. Serious side effects were very rare, with an incidence of migration and gastric perforation of 1.4 and 0.1%, respectively [11]. A large multicenter database analysis found a mean weight loss of 9.2 kg after 6 months and improvements of 5.1 kg after 3 years [12]. Nevertheless, in this analysis patients were included who had received additional therapy (lapband, gastric bypass or sleeve gastrectomy) after explantation of the balloons. In addition, many patients benefit from IB beyond explantation and relevant comorbidities such as arterial hypertension, diabetes or dyslipidemia were improved in about 50% of subjects [9, 13]. Nevertheless, although patients with IB reached a weight loss as high as 17.8 kg (> 10% of baseline) after 1 year in nonrandomized trials [14], some double-blinded, randomized, sham-controlled trials failed to show superiority of this device. Moreover, many patients did not achieve the goal of > 25% excess weight loss (EWL) or > 10% of baseline weight [15–17] and evidence of long-term effects is lacking [14]. Therefore, other endoscopic devices and strategies for the therapy of obesity have been developed.

The duodenal-jejunal bypass liner (DJBL) is an impermeable, fluoropolymer tube reversibly anchored to the duodenal bulb. Thereby, the chyme passes through the liner, while pancreatic and bile fluid passes outside the tube, resulting in an effective prevention of digestion and absorption in the upper intestine. The DJBL also has restrictive effects due to limited diameter of the sleeve and decelerated gastric emptying [18]. DJBL was originally developed for therapy of diabetes but is also known to induce considerable EWL of 19% [19] or 8.2 kg (EWL 11.9, 62% of participants achieved > 10% EWL) [20] up to 22.1 kg (EWL 47%) [21] after one year. In a six month follow-up after removal of the DJBL, some patients showed weight regain but continued to have a significantly higher weight loss compared to sham group [22]. Nevertheless, in analysis of both DJBL and IB, most studies evaluated excess weight loss (> 25% EWL) for definition of successful weight reduction. However, EWL strongly depends on baseline BMI and thus has unwanted properties [23, 24].

DJBL also improved glycemic control in obese patients with diabetes, and HbA1c was reduced from 8.7 ± 0.9% to 7.5 ± 1.6% [25]. These results were confirmed by several studies and a large meta-analysis [11, 18, 21, 26–29]. Furthermore, data indicating influence of DJBL on incretin hormone regulation and amelioration of fatty liver disease exist but remains preliminary [22, 26, 30, 31].

Although both devices were safe and showed mostly minor gastrointestinal complaints [7], a large multicenter study was dropped due to a high incidence of liver abscesses (3.5%) and an 11.7% rate of adverse events requiring removal of the DJBL (NCT01728116). In contrast, other studies did not observe such high rates of complications. In detail, serious adverse events in DJBL included migration (4.9%), GI bleeding (3.86%), sleeve obstruction (3.4%), liver abscess (0.126%), cholangitis (0.126%), acute cholecystitis (0.126%), and esophageal perforation (0.126%) secondary to trauma from an uncovered withdrawal [11].

In spite of convincing weight loss in patients undergoing endoscopic bariatric procedures, randomized studies comparing IB and DJBL are lacking. Thus, we designed a multi-center, randomized, controlled trial to compare weight loss in endoscopically implanted DJBL vs. IB vs. sham procedures. The weight-loss-endoscopy-trial (WET) aims to show the superiority of DJBL and IB compared with a sham group and to compare these devices. Additionally, the influence of DJBL and IB on diabetes and other comorbidities will be analyzed.

## Methods / design

### Trial organization and coordination

WET is designed and coordinated by the Division of Gastroenterology and Rheumatology together with the Clinical Trial Center, both at the University of Leipzig. WET will be conducted as a multi-center trial in Germany including the Sachsenhausen Clinic in Frankfurt, Bogenhausen Clinic in Munich, University Medical Center in Freiburg and University Medical Center Hamburg-Eppendorf in Hamburg.

The trial is sponsored by the German Research Foundation (DFG), which is not involved in the database management and has no access to randomization codes.

### Investigators

Patients will be recruited by the participating centers. All centers committed their participation in a covenant agreement with the trial coordinating institution. All investigators are experienced gastroenterologists and endoscopists and are certified as trial investigators who have attended additional courses on conducting trials according to the medical devices law.

### Data safety and monitoring board

An independent data safety and monitoring board consisting of three independent experts from the fields of gastroenterology, endocrinology and medical statistics will evaluate and supervise the clinical research data to assure patient safety and study integrity. The board will monitor the trial data, in particular the safety data and give their advice based on the periodical reviews.

### Medical device supply

The following medical devices will be used in this study. The EndoBarrier® system (endoscopically implanted DJBL, GI Dynamics, Duesseldorf, Germany, CE010311) and the Orbera Intragastric Balloon™ (Apollo Endosurgery, San Diego, USA, CE27493) are provided non-commercially by the study center. The DJBL will be implanted over a period of 12 months and the IB for a period of 6 months.

### On-site monitoring

On-site monitoring of the centers will be performed according to good clinical practice (ICH-GCP) guidelines. Personal visits will be carried out according to the SOPs of the Clinical Trial Center at Leipzig University. Clinical monitors will review entries into patient files (CRFs) on the basis of source documents (minimum of 30% source data verification). Monitors will verify source data and compliance with the procedures laid out in the trial protocol.

### Ethical considerations

The final study protocol was approved by the ethics committee of the Medical Faculty of the University of Leipzig (Trial registration number: DRKS00011036 on DRKS at 16th January 2017) in accordance with the declaration of Helsinki, the “Medical Association’s Professional Code of Conduct” and the principles of ICH-GCP guidelines (issued in June 1996, ISO14155 from 2012). In addition, the German Medical Devices Act (MPG, §§ 20-23a) was followed. Furthermore, local legal and regulatory authorities as well as the medical secrecy and the Federal Data Protection Act will be followed. All local ethics committees of the participating centers consented to the master ethics committee approval. Prior to enrollment, each patient will be given detailed information about the aims, scope and possible consequences of the trial by a physician. No diagnostic or interventional procedures required for the clinical trial will be performed without obtaining written consent from the patient.

### Study objectives

The primary objective of this study is to compare success rates (defined as ≥10% weight reduction from baseline weight) between the three procedures one year after removal. The primary analysis will use a generalized linear mixed model (GLMM) and a closed-testing procedure, meaning the three pairwise comparisons can be made without adjustment of the significance level if the global test is significant. Secondary aims include percentage weight loss at 12 months and success rates as well as percentage weight loss at 6 months and upon removal of the devices. In addition, complication rates and safety will be examined. Changes in obesity-related comorbidities (diabetes and associated diseases, hypertension, dyslipidemia, renal failure, coronary artery disease, heart failure, endocrinologic and psychiatric disorders, fatty liver and pulmonary diseases) and quality of life will also be assessed.

### Study design and setting

**Fig. 1:**
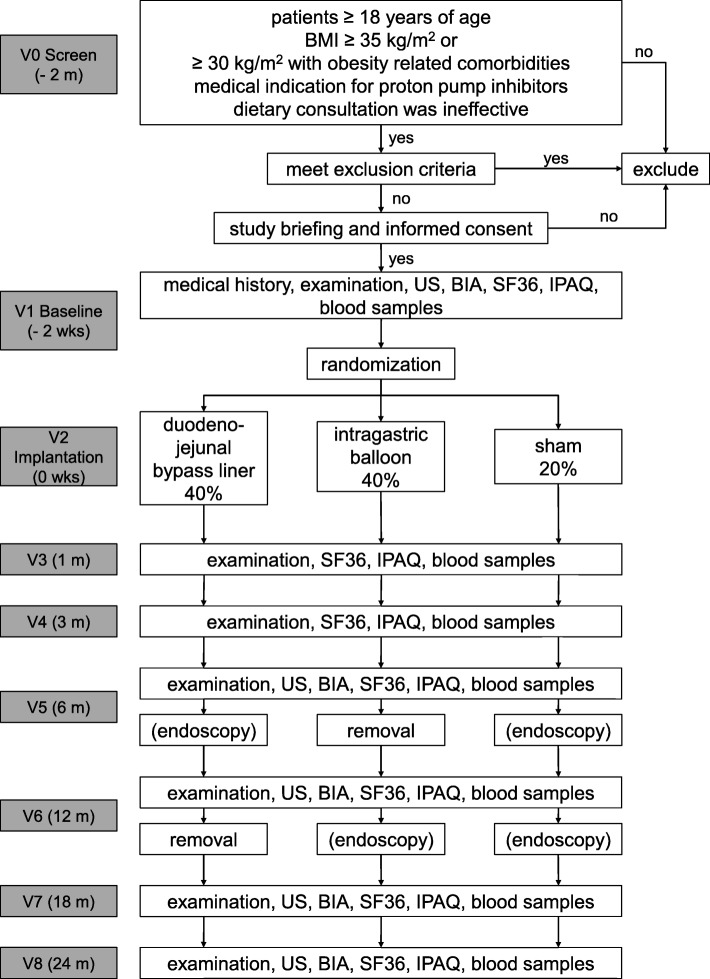
Treatment scheme

WET is a prospective, controlled, patient and assessor-blinded multi-center efficacy trial with two intervention arms and a sham procedure control arm. Patients will be randomized in a ratio of 2:2:1 to receive either IB, DJBL or sham procedure (gastroscopy with sedation). All enrolled patients will receive endoscopic procedures to implantat the devices or to maintain blinding. After 6 months, only the IB group, and after 12 months, only the DJBL group, will receive additional endoscopy to achieve explantation of the devices. The sham group will undergo sedation without gastroscopy at these same points in time. The duration of the trial for every patient is 24 months. The flowchart summarizing the trial visits is shown in Fig. 1.

Each site is required to have an endoscopy unit which meets all German quality standards. The possibility to perform endoscopies under fluoroscopy is mandatory. All participating centers will perform endoscopies under conscious sedation or general anesthesia with qualified anesthetists. All sites will appoint blinded and unblinded investigators and all unblinded investigators are experienced endoscopists. They are trained in the implantation procedure of the DJBL device in pigs and have independently implanted at least 10 DJBLs and 10 IBs. The blinded investigator will attend the patient at all study visits and will be in charge of all patient contacts except the endoscopies, thus ensuring the assessor blinded nature of the study.

### Patients

In all participating sites, adult patients (≥ 18 years of age) with a BMI ≥ 35 kg/m^2^ or ≥ 30 kg/m^2^ and obesity related comorbidities, will be screened for eligibility for the trial. Obesity-related comorbidities comprise arterial hypertension, diabetes and associated diseases, cardiovascular diseases, dyslipidemia, arthrosis, obstructive sleep apnea as well as endocrinologic, psychiatric and gastrointestinal diseases.

Prior to enrollment, all participants attempted conservative weight-loss therapy that was ineffective. Furthermore, all patients must have a medical indication for the long term use of proton pump inhibitors (PPI), due to the fact that PPI are mandatory for the implantation of DJBLs and could affect the primary endpoint differently from placebo. Exclusion criteria for the study are listed in Table 1. Additionally, a gastroduodenoscopy has to be performed in all participants within three months before recruitment to exclude gastric or duodenal ulcer or a large hiatal hernia.

**Table 1:** Exclusion criteria

Exclusion criteria	
Malignant disease	
Peptic ulcer	
Type 1 diabetes	
Large hiatus hernia	
Gastrointestinal diseases	
Previous gastric or bariatric surgery or endoscopic procedures (e.g. gastric balloon)	
High risk of gastrointestinal bleeding	
Symptomatic gallstones	
Contraindication for general anaesthesia	
Contraindication for devices according to the manufacturer	
Contraindication for proton pump inhibitors	
Non controlled gastrointestinal reflux disease	
Suspected lack of compliance	
Drug or alcohol abuse	
Pregnant or nursing women	
Fertile women without appropriate contraceptive measures while participating in the trial	
Participation in other interventional trials	
Psychological / mental or other inabilities to supply required information	

### Sample size considerations

The primary end point for the trial is successful weight loss 12 months after device removal. Removal is defined to be the true time of removal, if a device is explanted, or the date of endoscopy at 6 months, as will be the case for the sham group. Successful weight loss is defined to be a loss of at least 10% of the baseline weight. Different definitions of success have been recommended including excess weight loss [11]. However, EWL strongly depends on baseline BMI and thereby has considerable inaccuracies [24].

We estimated proportions of weight loss and the devices’ failures rates based on previously published work [12, 14–17, 19, 21]. At the end of the intervention, we anticipate 13.6, 8.3 and 6.1% weight loss for the completers in the DJBL, IB and sham arms, respectively. We assume a regain of 74% in the year follow-up for all three arms leading to estimates of 10.4, 6 and 3.9% weight loss for the “completers” of DJBL, IB and sham. After taking into account the expected failure rates of 15% (DJBL) and 10% (IB) and a drop-out rate of 10%, we estimate weight loss of 7.8, 4.8 and 3.5%, respectively. With a standard deviation of 4.8%, we expect successful weight reduction in 40, 16 and 9% of the respective participants.

Simulations show that at the 5% significance level, one has a power of 93% for the global test, 88% for the comparison between DJBL and sham and 84% between DJBL and IB with 150 patients. The secondary outcome of percentage weight loss would then have 89% power for the global test, 88% for the comparison between DJBL and sham and 75% between DJBL and IB. The width of the confidence interval for this last comparison would nonetheless be quite narrow at 4.3 percentage points and thus close to the limit of what can be considered a clinically relevant difference of 4 percentage points [32].

Regarding loss of follow up, compliance in the traditional sense is not an issue in this trial. The endoscopic devices cannot be explanted by the patient and any deviations from the recommended diet are part of the “real world” behavior that should be taken into account in the trial. On the other hand, problems with implantation and device failure take the place of compliance and are expected to be an issue. The failure estimates in the available literature vary wildly in the case of the DJBL from 0/16 (0%) over the course of one year [25] to 12/25 (48%) over the course of 12 weeks [20] and it was necessary to base our estimates of 15% for the DJBL and 10% for the IB on experience from our own university hospital. Loss to follow-up after removal is difficult to estimate because of the lack of data for long-term follow-up in comparable populations, yet we estimate a conservative 10%.

### Blinding and randomization

The study will be conducted in a patient and assessor-blinded fashion. Upon recruitment, each patient will receive a unique identification number to ensure both blinding of the patient and study team as well as identification after the end of the trial. Only the unblinded study team (implanters) will keep a personal list of patient numbers and names to match to patient records. After patients’ written informed consent has been obtained, the patient will be randomly assigned (1:2:2) either to the sham group (endoscopy in sedation) or one of the interventional groups (DJBL or IB), stratified with respect to diabetes status (yes/no) and center. The randomization will be performed electronically and every study participant can be identified by an ongoing unique randomization number. Randomization is organized and performed by the Center for Clinical Studies at the University of Leipzig and reported to the PI-center by fax within 24 h. The randomization codes are kept under lock and control, and they will be accessible 24 h a day in case of emergency code break.

### Statistical analysis

The primary end point for the trial is successful weight loss 12 months after removal. Successful weight loss is defined to be W_12month_/W_Baseline_ ≤ 0.9. The probability of successful weight loss per randomization arm, p_(success)_, is estimated to be the proportion of patients in that arm with successful weight loss. The null hypothesis of the trial is p_DBJL_(success) = p_IB_(success) = p_sham_(success). As mentioned above, primary and secondary study objectives will be analyzed by using a generalized linear mixed model (GLMM), which can take into account the longitudinal structure of the data as well as missing data. The stratification attribute diabetes will be included as a covariate in the model and a closed-testing procedure will be used. Thus, the three pair-wise tests will be performed if the global test is significant, where adjustment for the significance level is then unnecessary. Odds ratios and absolute differences in proportions along with confidence intervals based on the logistic regression will be presented. A sensitivity analysis will be performed in which the missing primary outcome will be treated as a failure, since dropping out is often rooted in poor weight loss. As a further sensitivity analysis, the 2 × 3 contingency table will be analyzed with a chi-squared test. A final sensitivity analysis will use the imputation results from the analysis of the continuous variable to define success/failure. Secondary analyses of weight as a continuous parameter will use a linear mixed model, but otherwise follow the lines of the primary analysis. As a sensitivity analysis, multiple imputation will be performed. Tests are all two-sided and the significance level is set at 5%.

The final analysis will be performed after the last patient has terminated the trial, and no interim analyses are planned.

### Treatment scheme

All recruited patients fulfilling inclusion criteria and under therapy with PPI will be randomized to receive sham endoscopy, DJBL or IB implantation. Sham endoscopy and IB will be performed under sedation whereas DJBL has to be implanted under general anesthesia. To maintain blinding, all participants will receive anesthetic informed consent and anesthesia will be performed after intravenous sedation prior to intubation. The sham group will always receive conscious sedation but will not be told that the choice of anesthetic is related to the study arm. The implantation of both devices or the initial sham endoscopy and the explantation after 12 months (DJBL) will be carried out as inpatient procedures. The following endoscopies after 6 months are planned to be outpatient examinations.

All patients will receive regular dietary consultations, clinical examinations, anthropometry, taking of blood samples for analysis of comorbidities (e.g. fasting blood glucose, HbA1c, lipids, inflammatory markers) and bioelectric impedance measurement (BIA). To evaluate hepatic abscesses as might occur as a complication of DJBL, periodic abdominal sonography (US) will be conducted. Furthermore, study patients will be evaluated for concomitant medication, obesity related comorbidities and adverse events or side effects. In addition, participants will complete the SF36 quality of life questionnaire at frequent intervals (see Fig. 1 for detailed trial flow chart). To conduct the above mentioned examinations, all patients are requested to attend study visits 1, 3, 6, 12 and 24 months after implantation of the devices or initial sham endoscopy. The study participation will be completed 24 months after implantation or baseline endoscopy. In the event of premature explantation of the device (e.g. due to abdominal pain or other complications), patients will be observed for an additional 12 months. To ensure blinding of the study, only the unblinded study team is permitted to perform the explantation.

Furthermore, the success of patient and assessor blinding will be analyzed and presented.

### Safety considerations

The safety of this trial will be evaluated based on the frequency of adverse events (AEs) and serious adverse events (SAEs) defined in DIN EN ISO 14155:11, section “Discussion”.2 and section “Discussion”.37. In addition, adverse device events (ADEs), serious adverse device effects (SADEs) and unanticipated serious adverse device events (USADEs) will be determined according to Medical Devices Safety Regulation §2. All AEs will be summarized and presented for the whole study group and each individual patient. The most common AEs (occurring in at least 10% of the appropriate group) will be determined. Laboratory data will be summarized by presenting summary and changes from baseline values (means, medians, standard deviations, ranges). The analysis of safety and tolerability will be based on all patients entered either into the DJBL or IB arm who received implantation of a device.

So far, a recent overview and meta-analysis showed overall safety and SAEs to be less than 5% in both devices [11]. Minor complications were not yet reported due to inconsistency of existing studies [14]. The sham group will receive conscious sedation, for which the risk of complication is known to be very low. The possible risks associated with sedation are further reduced by the use of only small doses of sedatives that are required to ensure blinding in the sham group.

## Discussion

Obesity is a global problem leading to associated cardiovascular and endocrinologic complications and reduced quality of life [33]. Conservative programs are often ineffective [4] whereas surgical procedures are irreversible and accompanied with serious complications as well as side effects and resources are unavailable for treating most patients [6]. Thus, endoscopic procedures present an important alternative. Previous studies showed both the feasibility and safety of DJBL and IB but revealed highly inconsistent data regarding effectiveness in weight loss. Only a few trials compared endoscopic interventions with sham procedures. Although some studies reported negligible weight loss in sham groups of 2–3 kg after 12 weeks [17, 20], others found a weight loss of as high as 12 kg as high as in the IB group [15]. These considerations lead to the necessity of a prospective, multi-center, randomized, controlled trial comparing weight loss in DJBL as compared to IB and sham group.

Thus, the WET trial aims to evaluate the superiority of DJBL and IB to sham group or each other in a patient and assessor-blinded setting. The primary objective of this study is weight loss ≥10% from baseline weight one year after removal of the devices. Secondary aims comprise proportion of weight loss, changes in obesity-related comorbidities and quality of life. Furthermore, complication rates of the devices and safety will be examined due to the fact that some studies showed an elevated risk of liver abscesses after implantation of DJBL [11].

The statistic calculation was carefully performed to ensure that the study is not underpowered. The novelty of our study design is that the WET study compares two endoscopic devices with different modes of action and different implantation periods related to the sham group. To ensure blinding of the study, all patients will undergo three endoscopies or sedation without gastroscopy, respectively. Although these requirements lead to a complex study protocol, the WET study will hopefully clarify important questions in endoscopic and conservative management of obesity.

## Conclusions

Assuming they are superior to a sham procedure, the more effective and safer device between DJBL and IB could become a more standard intervention for the therapy of obesity and its complications.

## Abbreviations

DJBL: duodenal-jejunal bypass liner
EWL: excess weight loss
IB: intragastric balloon
PPI: proton pump inhibitor
US: abdominal sonography
